# Knockdown of TOP2A suppresses IL‐17 signaling pathway and alleviates the progression of ulcerative colitis

**DOI:** 10.1002/iid3.1207

**Published:** 2024-04-25

**Authors:** Ou Li, Xuexiao Li, Jianping He

**Affiliations:** ^1^ Department of Proctology ZhuJiang Hospital of Southern Medical University Guangzhou Guangdong China

**Keywords:** bioinformatics, IL‐17 signaling pathway, TOP2A, ulcerative colitis

## Abstract

**Background:**

Ulcerative colitis (UC) is a chronic inflammatory disease of the colonic mucosa, with a gradually increasing incidence. Therefore, it is necessary to actively seek targets for the treatment of UC.

**Methods:**

Common differentially expressed genes (DEGs) were screened from two microarray data sets related to UC. Protein−protein interaction network was constructed to find the hub genes. The UC mouse model and cell model were induced by dextran sulfate sodium (DSS). The pathological changes of colon tissue were observed by hematoxylin‐eosin staining. Immunohistochemistry and immunofluorescence were performed to detect the expressions of Ki67 and Claudin‐1. The performance of mice was observed by disease activity index (DAI). The effect of TOP2A on proliferation, inflammation, oxidative stress, and interleukin‐17 (IL‐17) signaling pathway in UC model was measured by cell counting kit‐8, enzyme‐linked immunosorbent assay, and western blot.

**Results:**

Through bioinformatics analysis, 295 common DEGs were screened, and the hub gene TOP2A was selected. In UC model, there was obvious inflammatory cell infiltration in the colon and less goblet cells, while si‐TOP2A lessened it. More Ki67 positive cells and less Claudin‐1 positive cells were observed in UC model mice. Furthermore, knockdown of TOP2A increased the body weight and colon length of UC mice, while the DAI was decreased. Through in vivo and in vitro experiments, knockdown of TOP2A also inhibited inflammation and IL‐17 signaling pathway, and promoted proliferation in DSS‐induced NCM460 cells.

**Conclusion:**

Knockdown of TOP2A alleviated the progression of UC by suppressing inflammation and inhibited IL‐17 signaling pathway.

## Graphical abstract

Hub gene TOP2A was screened by bioinformatics analysis, which was associated with UC progression. Through in vivo and in vitro assays, Knockdown of TOP2A promoted proliferation and inhibited inflammation in UC, thereby alleviating UC development. Moreover, knockdown of TOP2A inhibited interleukin‐17 pathway in UC.

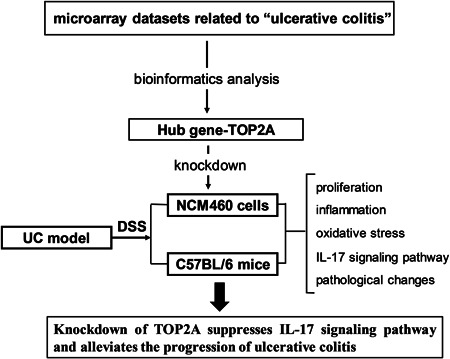

## 1 INTRODUCTION

Ulcerative colitis (UC) is a chronic inflammatory disease of the colonic mucosa that usually begins in the rectum and extends throughout the proximal part of the colon, often with bloody diarrhea.^1^, ^2^ The incidence of UC is gradually increasing due to its susceptibility to recurrence.^3^ In clinical practice, it is mainly diagnosed by endoscopy.^4^ Currently, medication is usually applied to alleviate UC and avoid complications, and in severe cases, surgical intervention is carried out.^2^, ^4^ Therefore, it is necessary to actively seek targets for the treatment of UC.

So far, several target genes have been evidenced to take part in the progression of UC. SLC6A14 promotes ferroptosis of epithelial cells through the C/EBPβ‐PAK6 axis of UC.^5^ Inhibition of miR‐222‐3p reduces oxidative damage to activate Nrf2/HO‐1 signaling by targeting BRG1, thereby improving UC and colorectal cancer.^6^ ACSF2 can mediate ferroptosis to participate in the progression of UC.^7^ Type II topoisomerases (TOP2) are able to catalyze DNA double‐strand breaks and are associated with a variety of dynamic processes such as transcription and cell division.^8^ TOP2 has two main paralogs in vertebrates, TOP2A and TOP2B.^9^ However, the role of TOP2A in UC is currently unknown.

Interleukin‐17 (IL‐17) is an important member of the new family of inflammatory cytokines, which is usually produced by unconventional T cells, innate lymphoid cells, and innate immune cells. It is significant in some chronic inflammatory diseases.^10^, ^11^ IL‐17 signaling pathway can induce acute immune defense in cells and contributes to wound tissue healing, barrier surface protection and repair.^12^ There is evidence that IL‐17 has a protective influence on the epithelial barrier of the colonic mucosa. Without IL‐17, the integrity of the intestinal epithelium will be damaged.^13^ In addition, Th17/IL17 can activate the NF‐KB/P53/Rb signaling pathway to induce endothelial cell senescence. At present, the effect of TOP2A on IL‐17 signaling pathway is still unclear.

In our study, bioinformatics analysis was applied to investigate the hub genes related to UC, and TOP2A was selected as the optimal indicator. Through in vitro and in vivo experiments, the roles of TOP2A in mouse performance, cell proliferation, inflammation, oxidative stress, and tight junction proteins were explored. In addition, due to the significance of IL‐17 signaling pathway in UC, the effect of TOP2A on IL‐17 pathway was also excavated, offering a new potential target for the therapy of UC.

## 2 MATERIALS AND METHODS

### 2.1 Microarray data sets

The gene expression profiles analyzed in this study were downloaded from the GEO database (https://www.ncbi.nlm.nih.gov/geo/) of the National Center for Biotechnology Information. Data sets related to “ulcerative colitis” (containing expression profiling from UC patients and healthy controls) were searched from the database, and two data sets, GSE9452, and GSE53306, were selected. The data sets were analyzed by GEO2R, and the differentially expressed genes (DEGs) (UC vs Control, noninflammatory bowel disease healthy controls) were identified in the GSE9452 and GSE53306 data sets according to *p* < .05 and |logFC | ≥1. The identified DEGs were visualized by volcano plots, and the samples of the data sets were standardized by box line plots with data corrections. In addition, Venn diagram and heat map of DEGs were drawn.

### 2.2 Functional analysis of common DEGs

After annotation and visualization, GO function enrichment analysis (biological process [BP], cellular component [CC], and molecular function [MF]) and KEGG enrichment analysis were performed on the screened overlapping DEGs by DAVID (https://david.ncifcrf.gov/summary.jsp). The GO‐KEGG data were obtained, and *p* < .05 was considered as statistical significance. GO and KEGG enrichment analysis of DEGs were analyzed by the R language, and the results with the most significant enrichment were displayed. Bar plots and bubble plots of enrichment analysis were shown.

### 2.3 Establishment of protein−protein interaction (PPI) network

DEGs were analyzed using the STRING (https://www.string-db.org/) to predict the interactions between proteins encoded by genes that may play a substantial role in the pathogenesis of colitis. For the significance criterion, the confidence interaction score was set at 0.700. Afterwards, the PPI network was visualized using Cytoscape (www.cytoscape.org/), and key modules were identified from the PPI network of DEGs using MCODE.

### 2.4 Analysis of hub genes

The enrichment chord plot and ridgeline plot of hub genes were drawn using R language. The expression of hub genes in the original sample data was selected as a variable for principal component analysis, and the principal component variables, PC1 and PC2, were obtained after processing with R. The correlation of hub genes in the expression profile matrix was visualized by drawing a matrix heat map. The receiver operating characteristic (ROC) curve was assessed by gene expression profile interaction analysis (http://gepia.cancer-pku.cn/), and the diagnostic accuracy of the selected genes was evaluated by the corresponding area under the ROC curve.

### 2.5 Construction of UC mouse model

Male C57BL/6 mice (6−8 weeks, 18−20 g) were acquired from SPF Biotechnology Co., Ltd. The mice were randomly grouped into four groups: Control, UC, LV‐NC, and LV‐TOP2A on average (*n* = 6). Lentivirus (1.0 × 10^9^ TU, 50 μL) encoding negative control shRNA and TOP2A shRNA were injected subcutaneously into the mice in the LV‐NC and LV‐TOP2A groups, respectively. The mice in the Control group drank water freely, and the mice in the other three groups drank 3% dextran sulfate sodium (DSS) (Sangon) freely to induce colitis for 7 days.

Body weight, fecal characteristics, and blood in feces were recorded daily and averaged to determine the disease activity index (DAI).^14^ The parameters were scored as follows: weight loss (0, <1%; 1, 1−5%; 2, 5−10%; 3, 10−15%, 4, >15%), presence or absence of blood in feces (0, negative; 2, feces concealing blood; 4, bloody), fecal consistency (0, negative; 2, loose stool; 4, watery stool). On the 8th day, mice were euthanized by carbon dioxide asphyxiation. Serum and colon tissues of mice were collected. In this study, all animal experiments conformed to the Guide for the Care and Use of Laboratory Animals, and have been approved by the Ethics Committee of ZhuJiang Hospital of Southern Medical University (LAEC‐2024‐011).

### 2.6 Hematoxylin‐eosin (HE) staining

Pathological changes of colon tissue were observed by HE staining. The colon tissue was fixed in 4% paraformaldehyde solution and dehydrated with an ethanol gradient on the next day. The tissue was transparent in xylene and embedded in paraffin solution. Subsequently, the tissue was cut into 5 μm thick sections for subsequent staining. Paraffin sections were dewaxed with conventional xylene and ethanol, and then stained with hematoxylin and eosin. The slices were dehydrated with ethanol, transparent with xylene, and sealed with neutral gum. The staining results were observed under CX43 optical microscope (Olympus).

### 2.7 Immunohistochemistry (IHC)

The expression of Ki67 in the colon tissue of mice was detected by IHC. The paraffin‐embedded sections were dewaxed and rehydrated, then incubated with anti‐Ki67 antibody (ab15580, 1/1000, Abcam) overnight at 4°C. Afterwards, the slices were then incubated with HRP‐labeled secondary antibody at 37°C for 1 h. Then the slices were stained with DAB and hematoxylin and finally photographed under CX43 optical microscope (Olympus).

### 2.8 Immunofluorescence (IF)

The expression of claudin‐1 in colon tissue of mice was detected by IF. The mouse colon tissue sections were dewaxed and then subjected to antigen repair at 92°C−96°C for 10−15 s, and naturally cooled to room temperature. Subsequently, the sections were blocked with normal goat serum at 37°C for 60 s, and then incubated with claudin‐1 antibody (ab211737, 1/1000, Abcam) at 4°C overnight. Afterwards, sections were incubated with Fluorescein‐labeled secondary antibody at 37°C for 60 s in the dark. Finally, the sections were sealed and observed by Leica TCS SPE laser scanning confocal microscope (Leica).

### 2.9 Enzyme‐linked immunosorbent assay (ELISA)

The levels of SOD, MDA, IL‐6, TNF‐α, and IL‐1β were detected using the corresponding ELISA kits (Esebio) following the manufacturer's instructions. Samples were added into each well and reacted with HRP‐labeled detection antibody, and then the plate was incubated for 60 min at 37°C. The liquid was removed, and the plate was washed. Substrates A and B were added, and the plate was incubated at 37°C for 15 min in the dark. A total of 50 μL of stopping solution was added to each well, and the OD value was measured at 450 nm within 15 min. The sample concentration was calculated by the standard curve.

### 2.10 Real‐time fluorescence quantitative PCR (RT‐qPCR)

TRIZOL reagent (Invitrogen) was utilized to extract total RNA. The concentration of RNA and the absorbance at 260 and 280 nm were measured by Evolution™ One/One Plus ultraviolet spectrophotometer (Thermo Fisher Scientific). The ratio of OD260/OD280 was between 1.9 and 2.0, indicating a high purity. The cDNA template was synthesized by Hiscript II QRT Supermix for qPCR (Vazyme). RT‐qPCR was performed using the ABI7500 quantitative PCR instrument (Applied Biosystems). The reaction conditions were: 95°C pre‐denaturation for 30 s, 40 cycles of 95°C denaturation for 10 s, and 60°C annealing for 30 s. GAPDH was chosen as the internal control. The obtained Ct value was analyzed by $2^{‐\Delta \Delta C_{t}}$ method. Primer sequences were shown in Table S1.

### 2.11 Cell culture and transfection

Human normal colonic epithelial cells NCM460 were cultured in DMEM (HyClone) containing 10% fetal bovine serum (Biosera) and 1% penicillin‐streptomycin (Beyotime). All cells were cultured at 37°C, 5% CO_2,_ and 95% humidity. To simulate UC in vitro, except for the control group, cells in other groups were treated with 2% DSS. siRNA targeting TOP2A (si‐TOP2A‐1,2,3) and siRNA negative control (siNC) were transfected into NCM460 cells by Lipofectamine 3000 (Thermo Fisher Scientific) (siRNA sequences were listed in Table S1).

### 2.12 Cell counting kit‐8 (CCK‐8) assay

CCK‐8 assay was used to detect cell viability. NCM460 cells (1 × 10^4^ cells/well) were incubated in 96‐well plates. After incubation for 24 h, 10 μL of CCK‐8 (Beyotime) was added, and then the cells were incubated for 2 h in the dark. The absorbance was read once every 1 h, and the optimal value was taken to determine the optimal culture time. Finally, the absorbance at 450 nm was determined by a microplate reader (Multiskan SkyHigh, Thermo Fisher Scientific).

### 2.13 Western blot (WB)

WB was exploited to detect the protein expression of ZO‐1, Occludin, IL‐17A, and IL‐17F in NCM460 cells. Proteins were extracted for SDS‐PAGE protein electrophoresis. Then the proteins were transferred to PVDF membrane at 4°C for 2 h. Samples were blocked with 5% skim milk TBST and shaken at room temperature for 2 h. The internal reference was GAPDH. Then antibodies (Abcam) were added, incubated at 4°C overnight with shaking, including ZO‐1 antibody (ab307799, 1/1000), Occludin antibody (ab216327, 1/1000), IL‐17A antibody (ab79056, 1/1000), and IL‐17F antibody (ab187059, 1/1000). Then, the membrane was washed and incubated with anti‐IgG secondary antibodies conjugated with horseradish peroxidase. Finally, ECL solution was added for developing, and images were captured with Tanon 5200 Chemiluminescent Imaging System (Tanon).

### 2.14 Statistical analysis

All statistical analyses were performed using GraphPad Prism 9.0. Data were expressed as mean ± standard deviation. One‐way ANOVA and Tukey's test were used for comparisons between multiple groups. *t*‐test was used for comparison between two groups. *p* < .05 were considered statistically significant.

## 3 RESULTS

### 3.1 Screening of DEGs

**Figure 1:**
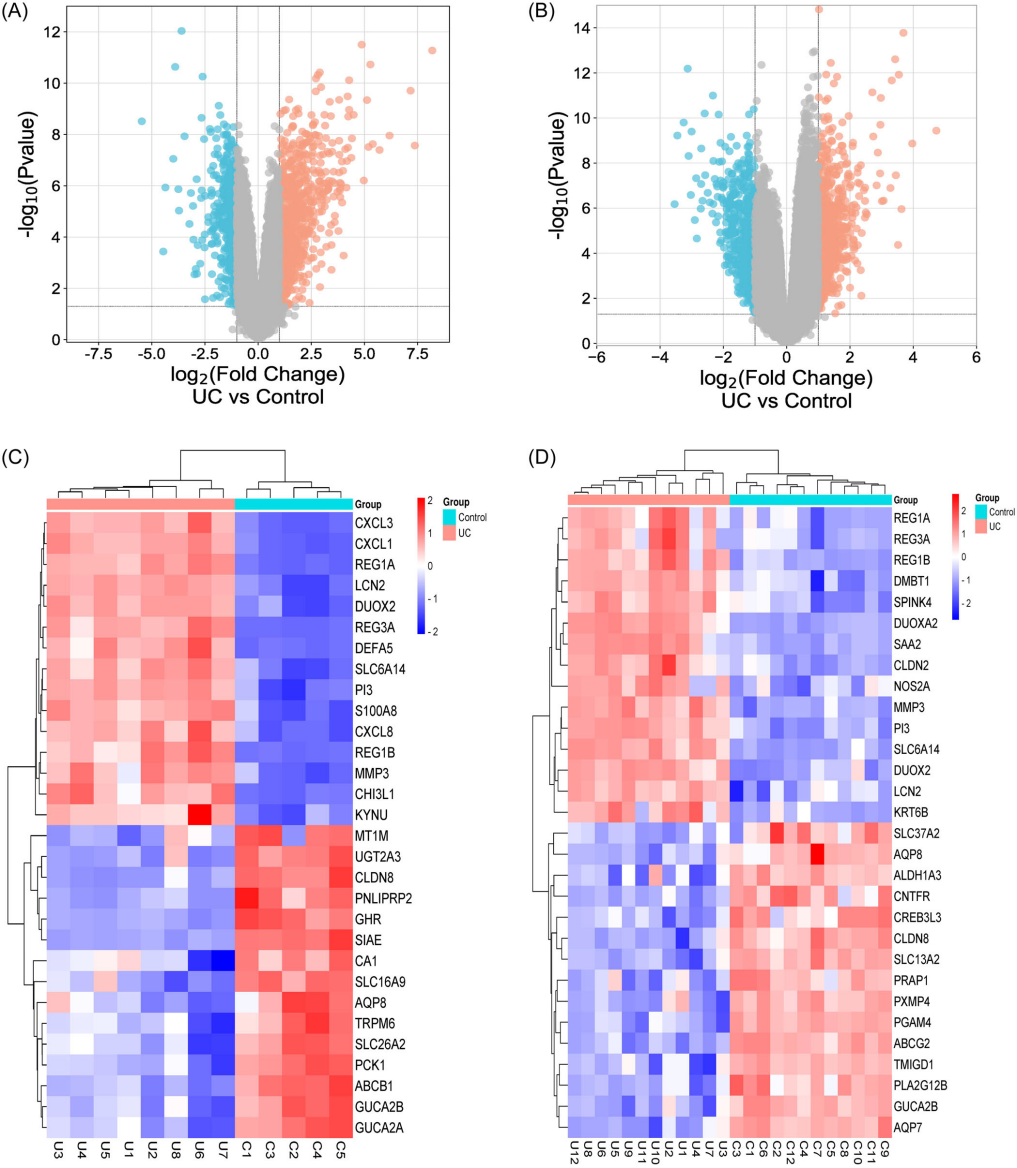
(A, B) Volcano plot of differentially expressed genes in the GSE9452 and GSE53306 data sets. Horizontal coordinates are log_2_FoldChange and vertical coordinates are ‐log_10_ (*p* Value). Red dots indicate upregulated genes in the group and blue dots indicate downregulated genes in the group. (C, D) Heat map of differentially expressed genes in the GSE9452 and GSE53306 data sets. Horizontal represents the gene, each column is a sample, red represents the high expression genes, blue represents the low expression genes.

Among the two data sets, there were five normal samples (Control) and eight colitis samples (UC) in GSE9452 data set, and 12 normal samples (Control) and 12 colitis samples (UC) in GSE53306 data set. A total of 1420 DEGs were screened from GSE9452, and 1479 DEGs were obtained from GSE53306. Cluster analysis was performed on these DEGs in the two data sets, and the volcano map was obtained (Figure 1A,B). After GEO2R analysis, the data correction results of 13 samples in GSE9452 data set and 24 samples in GSE53306 data set were obtained. According to the diagram of the two groups, the standardization and cross‐comparability of the data were evaluated. The results showed that most of the selected samples were centered, and the numerical distribution met the standard, indicating that the microarray data had a certain cross‐comparability (Figure S1). Subsequently, the top 15 DEGs in each data set were selected (Tables S2,S3), and visualized by R language (Figure 1C,D). According to the heat map of DEGs, the sample clustering is better and the confidence is higher. There were 295 common DEGs in the GSE9452 data set and the GSE53306 data set (Figure S2).

### 3.2 GO and KEGG pathway analysis

GO and KEGG functional analysis was performed on the 295 common DEGs. The top 6 GO term entries with the smallest p‐value in each GO classification were displayed (Figure S3A). DEGs were mainly enriched in the inflammatory response and extracellular matrix organization of BP, extracellular exosome and plasma membrane of CC, and extracellular matrix structural constituents and serine‐type endopeptidase activity of MF.

KEGG enrichment was measured by ‐log_10_(*p* Value), count value and the number of genes enriched to term. The top 15 KEGG Term entries with the smallest FDR value, that is, the most significant enrichment, were selected for display, showing that DEGs were mainly enriched in IL‐17 signaling pathway, Viral protein interaction with cytokine and cytokine receptors, and Pertussis (Figure S3B).

### 3.3 Identification of hub genes

A PPI network based on DEGs was constructed using STRING, and a highly interconnected cluster was identified as a potential functional molecular complex of colitis, including 8 hub genes, that is, NDC80, PBK, CEP55, RRM2, ASPM, NCAPG, TOP2A, and CDKN.

**Figure 2:**
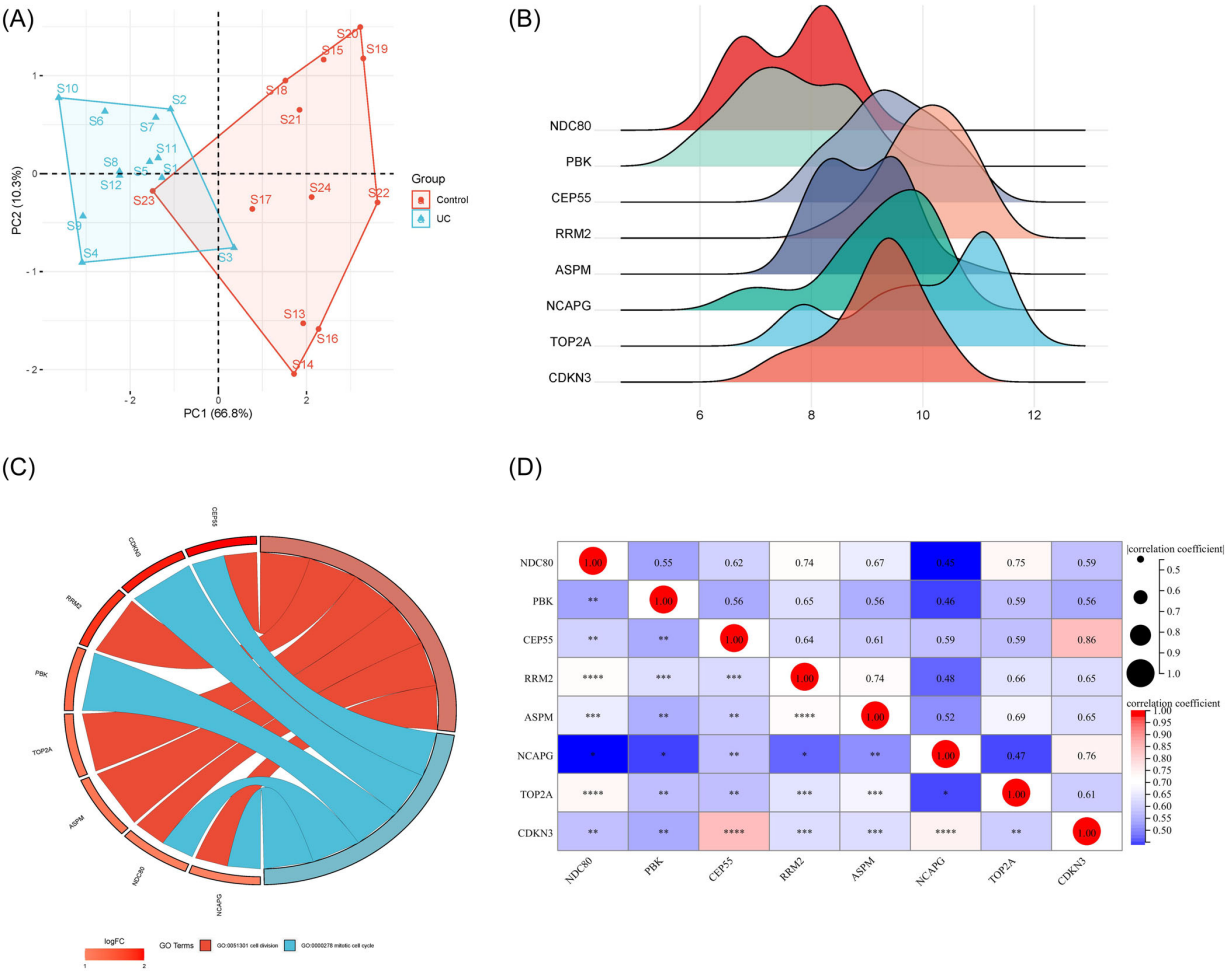
Analysis of hub genes. (A) Principal component analysis. The coordinate axes PC1 and PC2 in the graph are the first and second principal components (i.e., the explanatory rate of the latent variable to the difference). Points represent samples, and different colors represent different groups. (B) Ridgeline plot of hub genes. The abscissa is gene expression, the shape of the peak represents the dispersion between a set of data, and its height is the number of samples corresponding to the gene expression. (C) String diagram of hub genes. The data included 3 parts, genes, logFC and signaling pathways. LogFC is the fold change of the gene, which is used for sorting and gene block color. Different links of the gene indicate whether the gene is in the pathway. (D) Matrix correlation analysis of hub genes. Matrix analysis is used to perfectly display the correlation of genes between matrices and visualize them.

According to the expression of hub genes in the original sample data of GSE53306, principal component analysis was performed as a variable. After processing with R, PC1 and PC2 can provide a total explanation rate of 77.1% and can be used as a basis for distinguishing control samples from colitis samples (Figure 2A). The ridgeline plot and chord plot were drawn by R language, showing that DEGs were mainly enriched in cell division and mitotic cell cycle BPs (Figure 2B,C). In addition, to better show the relationship between proteins and pathways and indicate the changes in the function of pathways, a matrix heat map was drawn for visualization based on the expression profile data in GSE53306 data set (Figure 2D).

### 3.4 ROC curve

After literature research and screening, the key gene TOP2A was selected as the target gene. To verify the classification effect of TOP2A expression on the samples with colitis and healthy controls, the original sample data of GSE9452, and GSE53306 was used to draw the ROC curves of NDC80, PBK, CEP55, RRM2, ASPM, NCAPG, TOP2A and CDKN3 by R language (Figure S4). The results showed that the false positive rate was 0% and the true positive rate was 100% with TOP2A as the index based on GSE9452 as the original sample data, while the false positive rate was 3.5% and the true positive rate was 96.5% based on GSE53306 as the original sample data. It indicated that TOP2A can be used as one of the best indicators to distinguish between colitis and healthy controls.

### 3.5 Construction of UC mouse model

**Figure 3:**
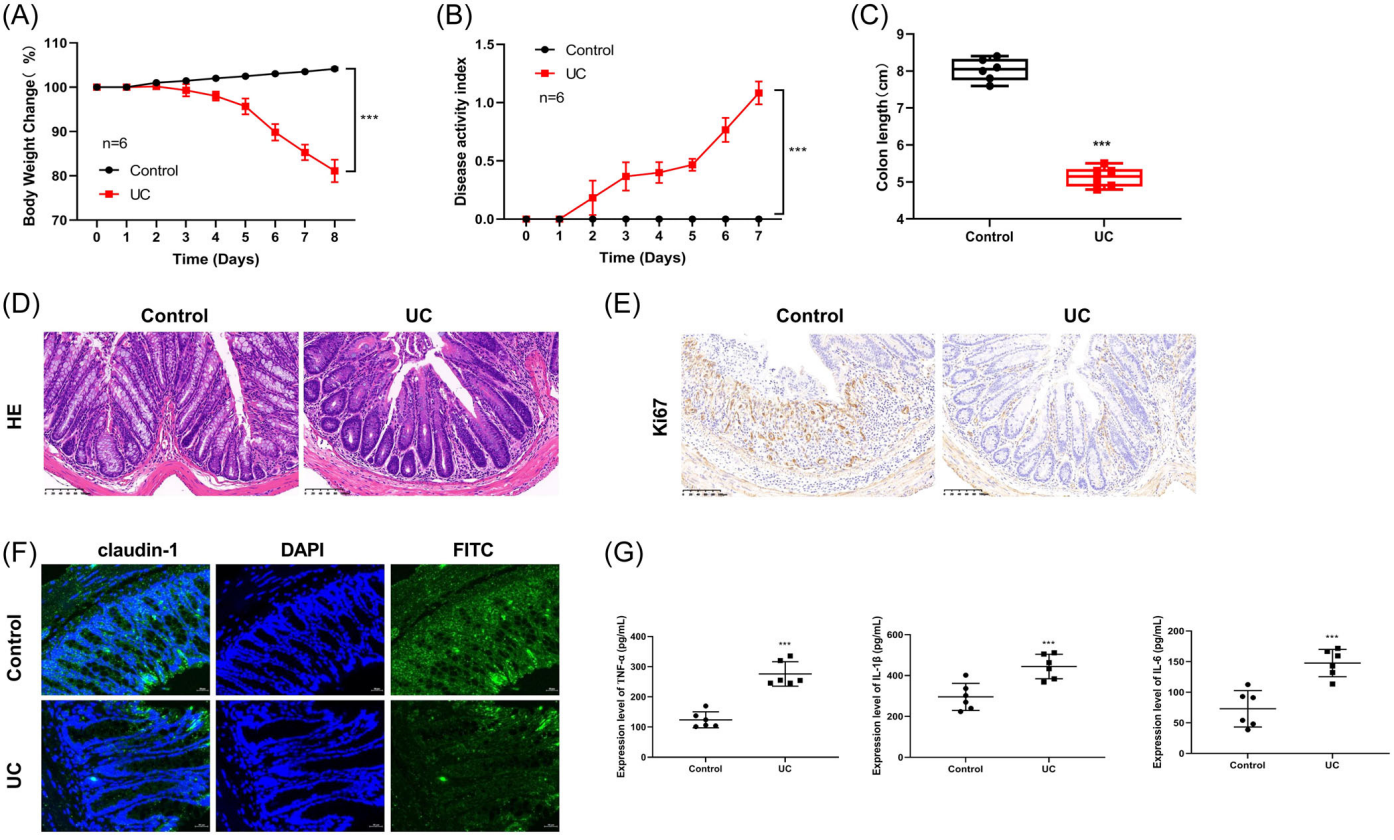
Physiological changes of UC model mice. (A) Weight changes of mice. (B) Disease activity index. (C) Mouse colon length. (D) Hematoxylin‐eosin staining was used to observe the histopathological changes (×200, 100 μm). (E) Immunohistochemical determination of Ki67 level (×200, 100 μm). (F) The expression of claudin‐1 protein was detected by immunofluorescence (×100, 20 μm). (G) The levels of TNF‐α, IL‐1β and IL‐6 in serum of mice in each group were determined by ELISA. **p < .01 ***p < .001 versus control.

In Comparison with the Control group, the body weight of mice in the UC group was significantly reduced (Figure 3A), and the DAI was significantly increased on the second day (Figure 3B). Meanwhile, the colon length of mice was significantly reduced in the UC group (Figure 3C).

HE staining showed that in the Control group, the colon tissue structure was clear, and the intact was epithelium, with abundant goblet cells and closely arranged cells, and there was no inflammatory cell infiltration. Compared with the Control group, there was obvious inflammatory cell infiltration in crypt in the UC group, the number of goblet cells was significantly reduced, and the villous structure was destroyed (Figure 3D).

IHC showed that compared with Control group, there was a significantly decreased positive Ki67 cells in the UC group (Figure 3E). IF was carried out to detect the expression of claudin‐1, demonstrating that positive claudin‐1 cells in the UC group were more than those in the Control group (Figure 3F). Moreover, the concentrations of inflammatory cytokines IL‐1β, IL‐6, and TNF‐α in the UC group were significantly increased (Figure 3G).

### 3.6 RT‐qPCR validation

**Figure 4:**
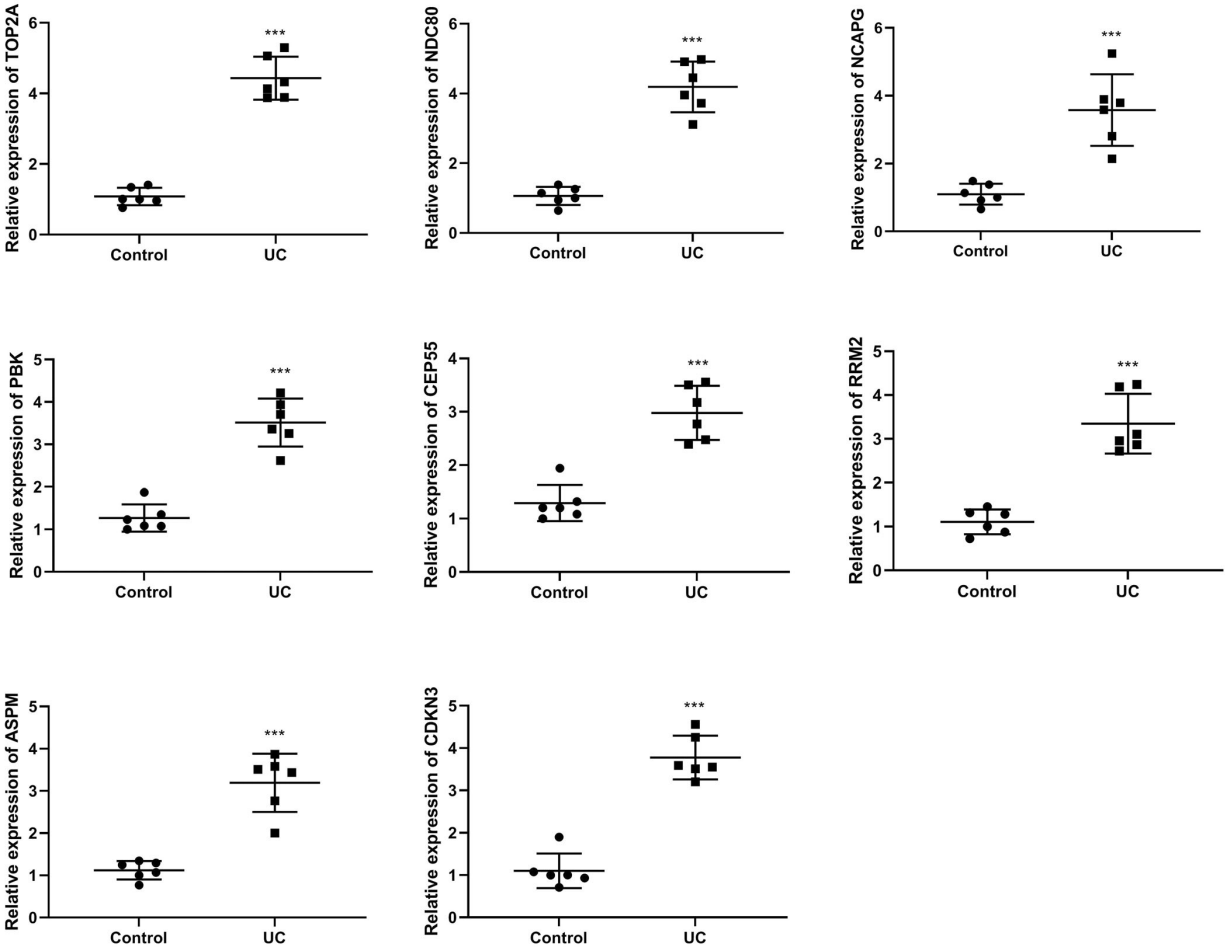
The mRNA expression levels of NDC80, PBK, CEP55, RRM2, ASPM, NCAPG, TOP2A, and CDKN3 were detected by RT‐qPCR. ***p < .001 versus control.

The mRNA expression levels of hub genes were verified by RT‐qPCR, showing that compared with the Control group, the expression levels of NDC80, PBK, CEP55, RRM2, ASPM, NCAPG, TOP2A and CDKN3 were significantly upregulated in the UC group (Figure 4).

### 3.7 Knockdown of TOP2A promotes proliferation and inhibits inflammation in DSS‐induced NCM460 cells

**Figure 5:**
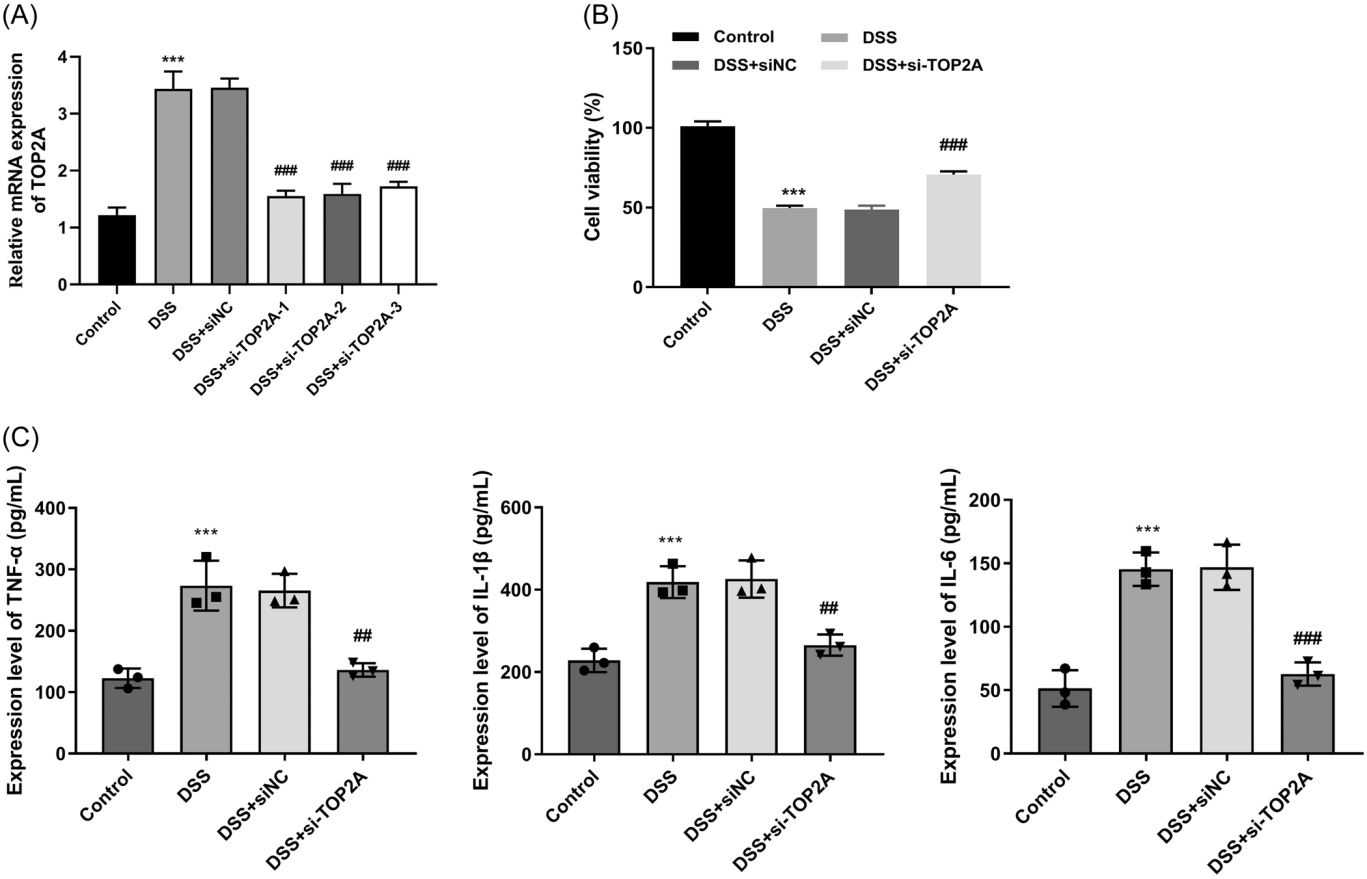
(A) RT‐qPCR was used to detect the mRNA expression level of TOP2A. (B) CCK‐8 assay was performed to evaluate cell viability. (C) ELISA was used to detect the content of inflammatory factors TNF‐α, IL‐1β and IL‐6. ***p < .001 versus control ^##^ *p* < .01 ^###^ *p* < .001 versus DSS + siNC.

In DSS‐induced NCM460 cells, the expression of TOP2A was upregulated. si‐TOP2A‐1, si‐TOP2A‐2, and si‐TOP2A‐3 all resulted in a significant downregulation of TOP2A expression, and si‐TOP2A‐1 with the best transfection efficiency was selected for the following experiments (Figure 5A).

The cell viability was detected by CCK‐8 assay, demonstrating that the cell viability of colon cells was significantly decreased after DSS treatment, and knockdown of TOP2A significantly increased the cell viability (Figure 5B).

ELISA showed that, compared with the Control group, the levels of IL‐1β, IL‐6, and TNF‐α in DSS‐induced NCM460 cells were significantly increased. Compared with the DSS + siNC group, the levels of IL‐1β, IL‐6 and TNF‐α were significantly reduced after knocking down TOP2A (Figure 5C).

### 3.8 Knockdown of TOP2A inhibits IL‐17 pathway in DSS‐induced NCM460 cells

**Figure 6:**
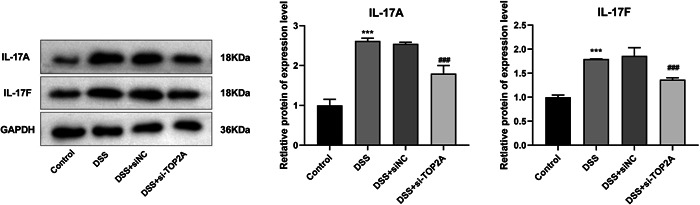
Western blot was used to verify the expression of IL‐17 pathway‐related proteins (IL‐17A, IL‐17F). ***p < .001 versus control ^##^ *p* < .01 ^###^ *p* < .001 versus DSS + siNC.

The key proteins of IL‐17 pathway were detected by WB, exhibiting that compared with the Control group, the protein expressions of IL‐17A and IL‐17F in the cells were increased after DSS treatment. After knocking down TOP2A, the protein expressions of IL‐17A and IL‐17F were significantly decreased compared with the DSS + siNC group (Figure 6), proving that TOP2A can regulate the IL‐17 pathway in UC.

### 3.9 Knockdown of TOP2A alleviates the progression of UC

**Figure 7:**
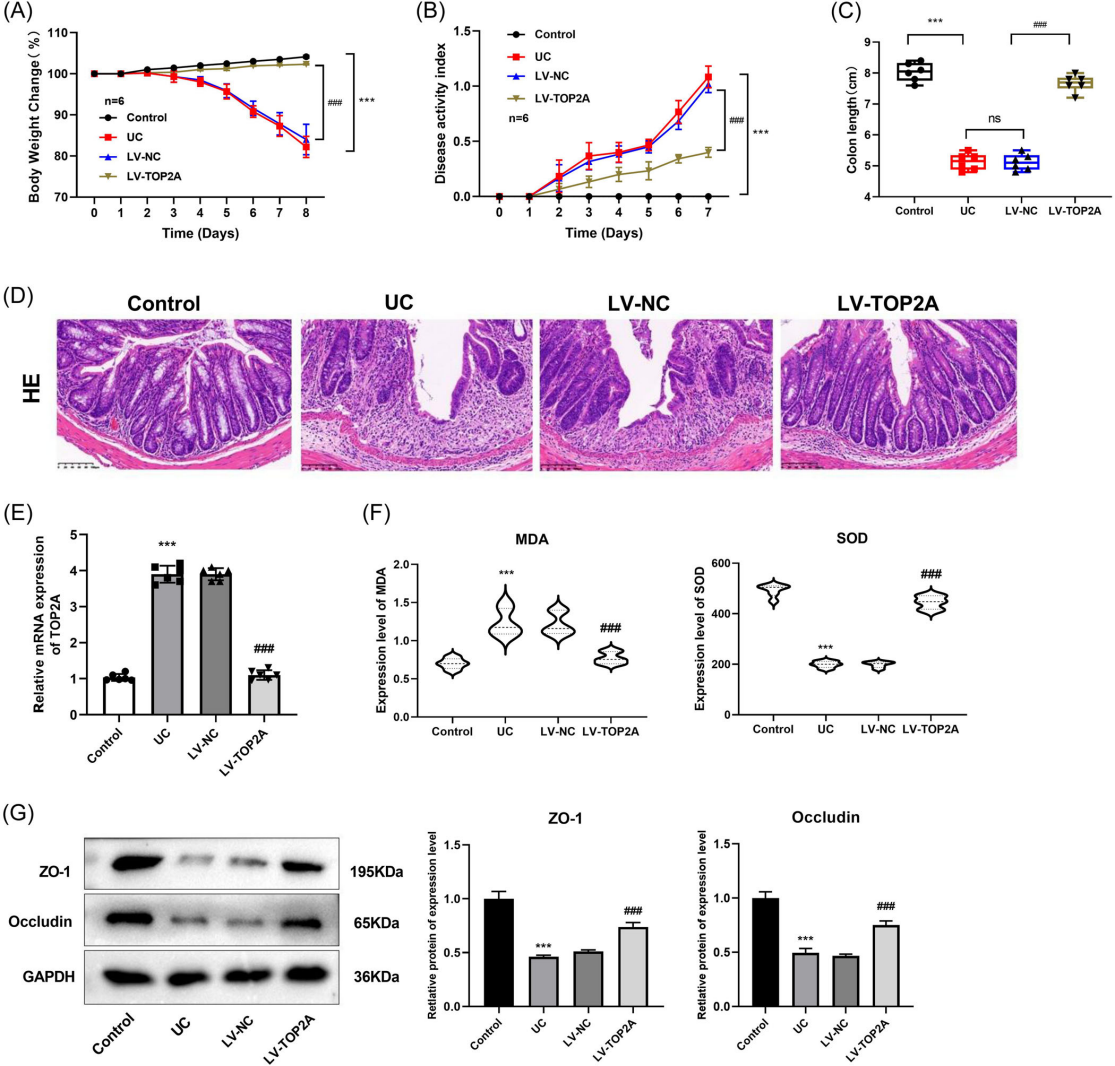
Effects of TOP2A knockdown on UC model mice. (A) Weight changes in mice. (B) Disease activity index. (C) Mouse colon length. (D) HE staining was used to observe the pathological changes of colon tissue. (E) qPCR was used to detect the expression of TOP2A in colon tissue. (F) The levels of oxidative stress indexes MDA and SOD in serum of mice were determined by ELISA. (G) Western blot was used to detect the expression levels of tight junction proteins ZO‐1 and Occludin in colon tissue. ***p < .001 versus Control ^##^ *p* < .01 ^###^ *p* < .001 versus LV‐NC.

To investigate the role of TOP2A on colitis in vivo, a knockout TOP2A mouse model of colitis was further constructed. The results showed that the body weight of mice in the LV‐TOP2A group was significantly increased compared with that of the LV‐NC group (Figure 7A), and the DAI of mice in the LV‐TOP2A group was significantly lower (Figure 7B), as well as an increased colon length (Figure 7C).

HE staining demonstrated that the colonic mucosa of mice in the Control group was intact. In DSS‐induced UC group, mucosal integrity was lost, and goblet cells were significantly reduced. Compared with the UC group, knockdown of TOP2A led to the increase in the goblet cell populations in colonic tissues, while inflammatory cell infiltration was significantly reduced (Figure 7D).

RT‐qPCR detected that the expression of TOP2A was significantly decreased in the colon tissue of mice in the LV‐TOP2A group compared with that in the LV‐NC group (Figure 7E).

ELISA results showed that the concentration of MDA, an indicator of oxidative stress, in the serum of mice in the UC group was significantly higher and the concentration of SOD was significantly lower compared with that of the Control group, and knockdown of TOP2A resulted in a significant decrease in MDA and a significant increase in SOD (Figure 7F).

WB showed that the expression levels of tight junction proteins ZO‐1 and Occludin in UC group were significantly decreased compared with the Control group, and the expression levels of ZO‐1 and Occludin protein were significantly elevated after knockdown of TOP2A (Figure 7G).

### 3.10 Knockdown of TOP2A inhibits IL‐17 pathway in UC mouse model

**Figure 8:**
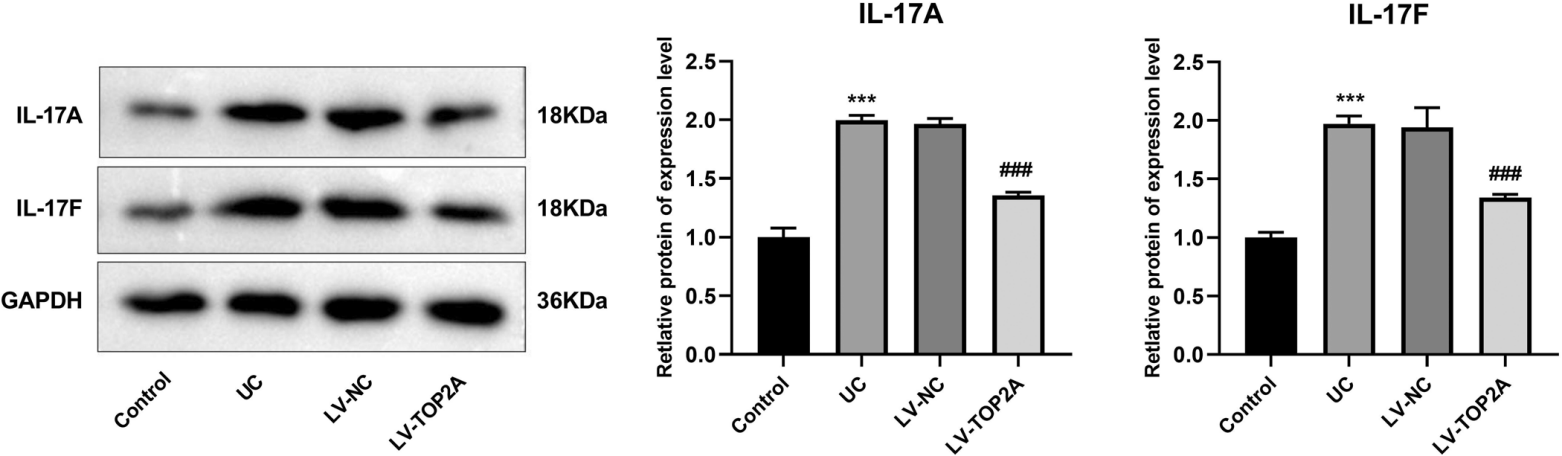
Western blot was used to verify the expression of IL‐17 pathway‐related proteins (IL‐17A, IL‐17F) in colon tissue. ***p < .001 versus control ^##^ *p* < .01 ^###^ *p* < .001 versus LV‐NC.

The key proteins of IL‐17 pathway were detected by WB, revealing that the protein expressions of IL‐17A and IL‐17F was elevated in the UC group compared with the Control group, and knockdown of TOP2A significantly reduced the expression of IL‐17A and IL‐17F compared with the LV‐NC group (Figure 8), which demonstrated that TOP2A could regulate the IL‐17 pathway in UC.

## 4 DISCUSSION

UC is an inflammatory disease of the colon, which may be life‐threatening in severe cases.^15^ Currently, the clinical treatment of UC is mainly to ameliorate the intestinal mucosal barrier and reduce the secretion of inflammatory factors.^16^, ^17^ In this paper, eight hub genes were selected by bioinformatics analysis, and the key gene TOP2A was selected for the following studies. The regulatory effect of TOP2A was verified through cell and mouse experiments. We found that knockdown of TOP2A significantly promoted cell proliferation, inhibited DAI of mice, inflammation, and oxidative stress in UC model, and suppressed IL‐17 signaling pathway. Furthermore, knockdown of TOP2A also improved the inhibition of tight junction protein expression in UC model. It was proved that knockdown of TOP2A inhibited inflammation and oxidative to alleviate the progression of UC, as well as the inhibition of IL‐17 signaling pathway, providing a potential target for the therapy of UC.

Through bioinformatics analysis, DC80, PBK, CEP55, RRM2, ASPM, NCAPG, TOP2A, and CDKN3 were screened, which seemed to be associated with the progression of UC. PBK is called PDZ Binding Kinase, and studies have demonstrated that PBK is an effective drug target with multiple therapeutic potentials in a variety of cancers, such as ovarian cancer, prolactinoma, and cervical cancer.^18^, ^19^, ^20^ Moreover, the inhibitor of PBK can suppress the proliferation of colorectal cancer cells with PBK expression.^21^ He et al. screen out 10 hub genes by bioinformatics analysis, including CEP55, which may be diagnostic biomarkers for UC.^22^ High expression of CEP55 has also been associated with poor prognosis of non‐small cell lung cancer, gallbladder cancer, and endometrial cancer.^23^, ^24^, ^25^ Chen et al. identify 14 key genes related to the pathogenesis of colon cancer, including CEP55, TOP2A, RRM2, NCAPG2, providing new directions for elucidating the molecular mechanisms of colon cancer.^26^ ASPM has also been shown to promote cancer progression.^27^, ^28^ TOP2A and CDNK3 have been screened to be involved in colorectal cancer progression.^29^

Currently, TOP2 has been widely used in targeted therapy of cancers. By targeting TOP2 to disrupt the catalytic cycle of TOP2, it triggers the DNA damage response and promotes apoptosis.^30^, ^31^ TOP2A may promote hepatocellular carcinoma progression by facilitating epithelial‐mesenchymal transition mediated by the p‐ERK1/2/p‐SMAD2/Snail pathway, which has been associated with a poor prognosis in hepatocellular carcinoma.^32^ In addition, TOP2A has been associated with poor prognosis in cancers such as medulloblastoma and lung adenocarcinoma.^33^, ^34^ In sepsis‐induced acute lung injury, overexpression of miR‐125b‐5p inhibits TOP2A to alleviate the disease.^35^ Herein, in DSS‐induced UC model, DAI of mice was increased, and the expression of inflammatory factors was promoted, while knockdown of TOP2A reversed it, demonstrating that knockdown of TOP2A can alleviate the progression of UC by inhibiting inflammation.

Studies have demonstrated the significance of IL‐17 in the progression of UC. In 2021, Novielo and his team reveal the pivotal role of the IL23/IL‐17 immune axis in the pathogenesis of UC.^36^ Evidence has reported that Andrographolide improves UC by the suppression of the IL‐23/IL‐17 axis.^37^ Hydroethanolic extract reduces colonic injury and attenuates oxidative damage in a TNBS model of colitis by increasing mucus production, decreasing leukocyte migration, and decreasing IL‐17, IL‐1β, TNF‐α and COX‐2 expression (in vivo and in vitro), reducing colonic damage and attenuating oxidation and inflammation in a TNBS model of colitis.^38^ In addition, miR‐155 targets Est‐1 and induces UC through the IL‐23/17/6‐mediated Th17 pathway.^39^ Fusobacterium nucleatum upregulates CARD17 expression to activate the IL‐17F signaling pathway, thereby promoting UC.^40^ In this study, si‐TOP2A significantly inhibited the IL‐17A, and IL‐17F, which indicated that knockdown of TOP2A can suppress IL‐17 signaling pathway in UC.

## 5 CONCLUSION

Collectively, through intro and in vivo experiments, it was verified that knockdown of TOP2A significantly promoted cell proliferation, inhibited inflammation, and oxidative stress to alleviate the progression of UC, as well as the inhibition of the IL‐17 signaling pathway, which provided a novel therapeutic target for UC.

## AUTHOR CONTRIBUTIONS

**Ou Li**: Conceptualization, data curation, methodology, formal analysis, writing‐original draft preparation. **Xuexiao Li**: Data curation, investigation, validation, resources, writing‐review and editing. **Jianping He**: Project administration, supervision, writing‐review and editing. all authors have read and approved the article. All listed authors should have contributed to the manuscript substantially and have agreed to the final submitted version.

## CONFLICT OF INTEREST STATEMENT

The authors declare no conflict of interest.

## ETHICS STATEMENT

All animal experiments conformed to the Guide for the Care and Use of Laboratory Animals, and have been approved by the Ethics Committee of ZhuJiang Hospital of Southern Medical University (LAEC‐2024‐011).

## Supporting information

Supporting information.

Supporting information.

Supporting information.

## Data Availability

The data that support the findings of this study are available from the corresponding author upon reasonable request.
