# Supplementary material for: Knockdown of TOP2A suppresses IL‐17 signaling pathway and alleviates the progression of ulcerative colitis
Source: Immun Inflamm Dis. 2024 Apr 25;12(4):e1207. doi: 10.1002/iid3.1207 (PMC11044219; doi:10.1002/iid3.1207)
Supplement: Supplementary file 1 — Supporting information. [file IID3-12-e1207-s002.pdf]

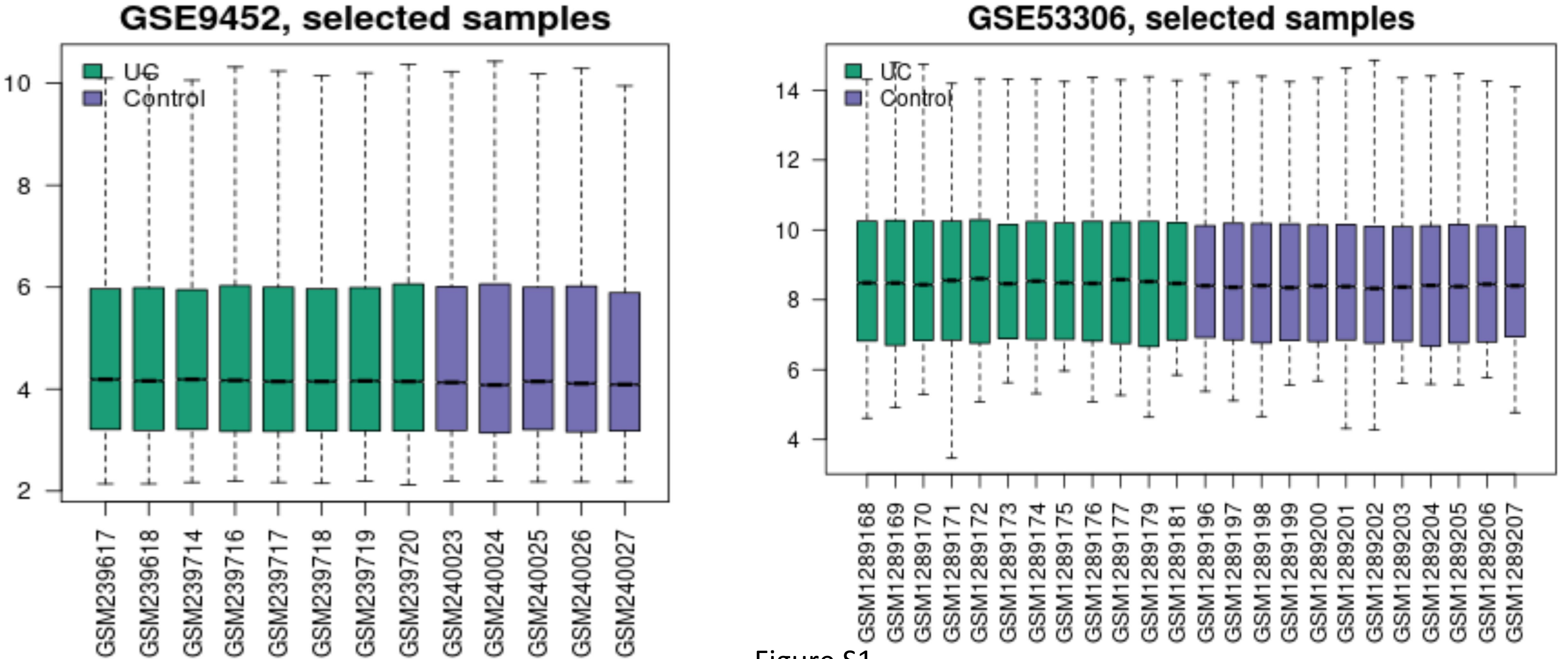
GSE9452, selected samples
UC
Control
10
8
6
4
2
GSM239617
GSM239618
GSM239714
GSM239716
GSM239717
GSM239718
GSM239719
GSM239720
GSM240023
GSM240024
GSM240025
GSM240026
GSM240027
GSE53306, selected samples
UC
Control
14
12
10
8
6
4
GSM1289168
GSM1289169
GSM1289170
GSM1289171
GSM1289172
GSM1289173
GSM1289174
GSM1289175
GSM1289176
GSM1289177
GSM1289179
GSM1289181
GSM1289196
GSM1289197
GSM1289198
GSM1289199
GSM1289200
GSM1289201
GSM1289202
GSM1289203
GSM1289204
GSM1289205
GSM1289206
GSM1289207

Figure S1

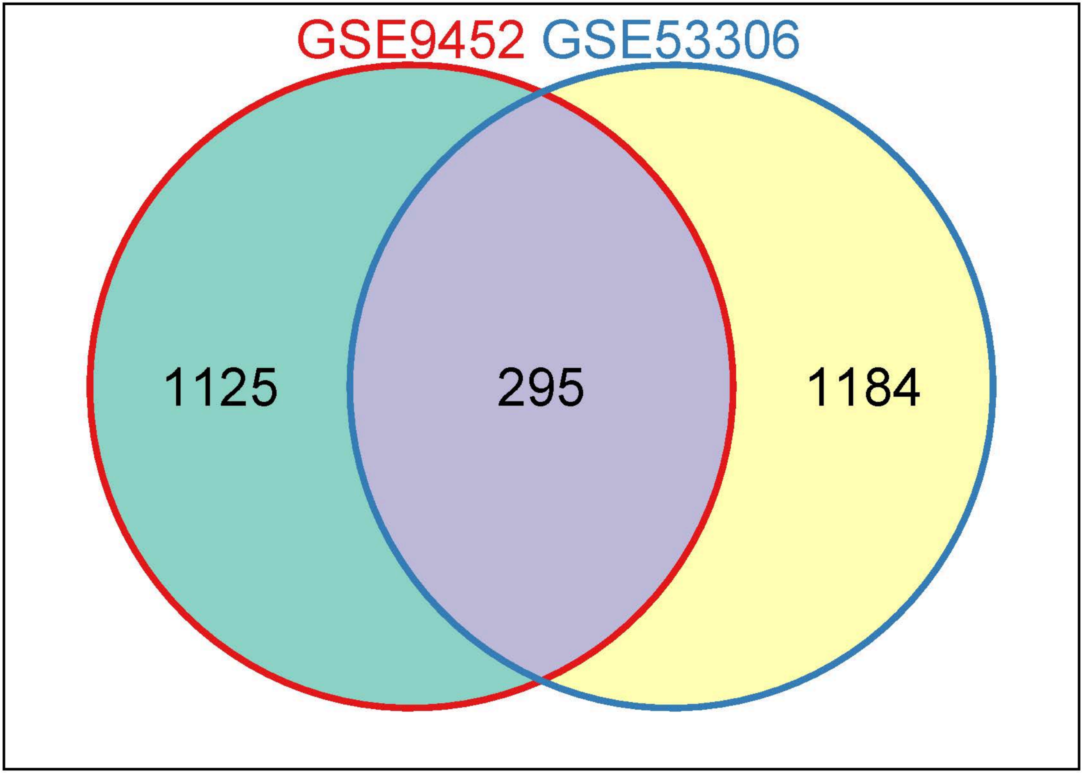


Figure S2

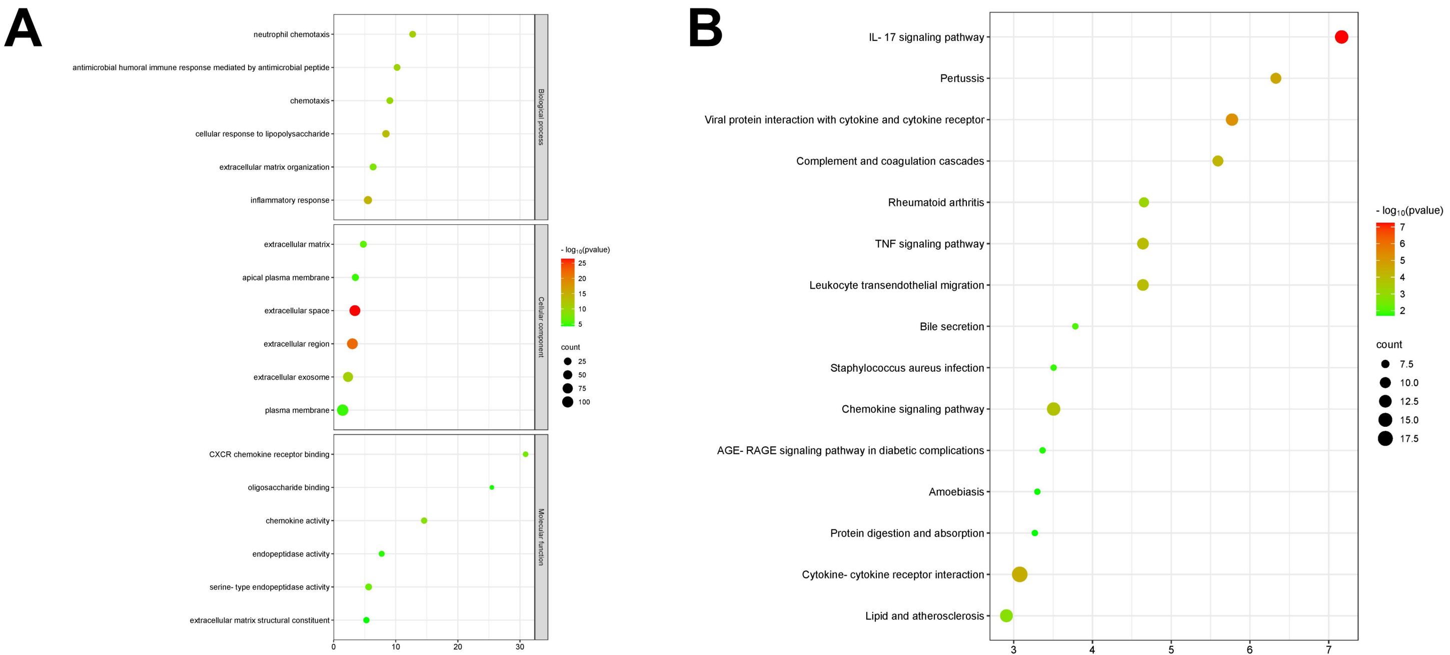
A
neutrophil chemotaxis
antimicrobial humoral immune response mediated by antimicrobial peptide
chemotaxis
cellular response to lipopolysaccharide
extracellular matrix organization
inflammatory response
extracellular matrix
apical plasma membrane
extracellular space
extracellular region
extracellular exosome
plasma membrane
CXCR chemokine receptor binding
oligosaccharide binding
chemokine activity
endopeptidase activity
serine- type endopeptidase activity
extracellular matrix structural constituent
Biological process
Cellular component
Molecular function
0
10
20
30
- log10(pvalue)
25
20
15
10
5
count
25
50
75
100
B
IL- 17 signaling pathway
Pertussis
Viral protein interaction with cytokine and cytokine receptor
Complement and coagulation cascades
Rheumatoid arthritis
TNF signaling pathway
Leukocyte transendothelial migration
Bile secretion
Staphylococcus aureus infection
Chemokine signaling pathway
AGE- RAGE signaling pathway in diabetic complications
Amoebiasis
Protein digestion and absorption
Cytokine- cytokine receptor interaction
Lipid and atherosclerosis
3
4
5
6
7
- log10(pvalue)
7
6
5
4
3
2
count
7.5
10.0
12.5
15.0
17.5

Figure S3

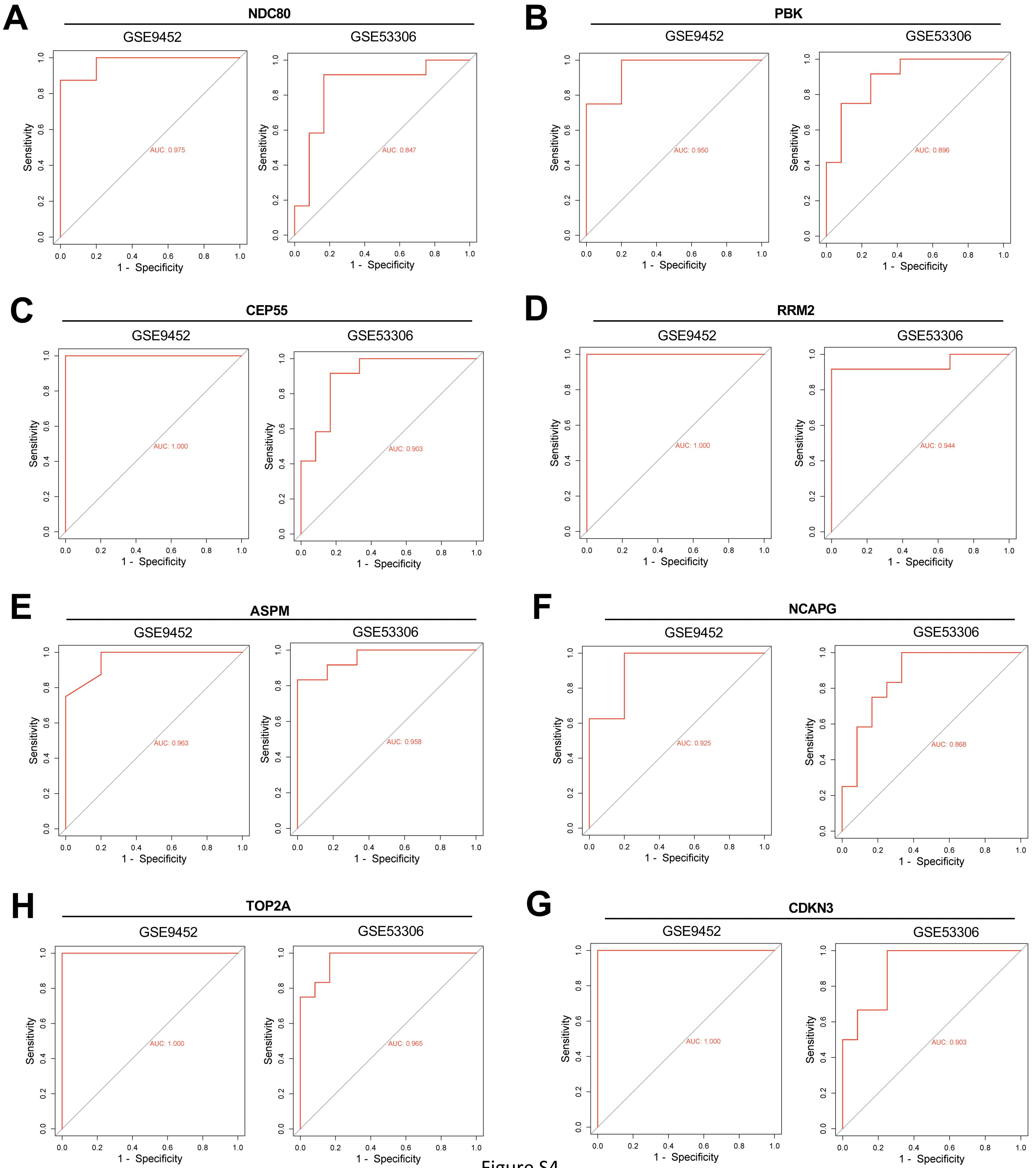
A
NDC80
GSE9452
GSE53306
AUC: 0.975
AUC: 0.847
B
PBK
AUC: 0.950
AUC: 0.896
C
CEP55
AUC: 1.000
AUC: 0.903
D
RRM2
AUC: 1.000
AUC: 0.944
E
ASPM
AUC: 0.963
AUC: 0.958
F
NCAPG
AUC: 0.925
AUC: 0.868
H
TOP2A
AUC: 1.000
AUC: 0.965
G
CDKN3
AUC: 1.000
AUC: 0.903
Sensitivity
1 - Specificity

Figure S4
