# Supplementary material for: Knockdown of TOP2A suppresses IL‐17 signaling pathway and alleviates the progression of ulcerative colitis
Source: Immun Inflamm Dis. 2024 Apr 25;12(4):e1207. doi: 10.1002/iid3.1207 (PMC11044219; doi:10.1002/iid3.1207)
Supplement: Supplementary file 2 — Supporting information. [file IID3-12-e1207-s003.docx]

**Table S1 Information of siRNA and primer sequences in this study**

| Name | 5’-3’ | 3’-5’ |
| --- | --- | --- |
| siNC | UUCUCCGAACGUGUCACGUTT | ACGUGACACGUUCGGAGAATT |
| si-TOP2A-1 | GCAAUUCUAUGCUGACAUAAC | UAUGUCAGCAUAGAAUUGCUG |
| si-TOP2A-2 | CCUGAUUCUGCAAAGAUAAAU | UAGUGCUUAUAGCUCUUCCUG |
| si-TOP2A-3 | GGAGAAGAUUAUACAUGUAUC | UACAUGUAUAAUCUUCUCCAU |
| TOP2A | AACAGCCTGTACCCTGATGG | CGGTAGTGCCCATCATTCTT |
| GAPDH | AACTTTGGCATTGTGGAAGG | ACACATTGGGGGTAGGAACA |
| NDC80 | TCCTCATACATGGCCTCACA | TGTCGGCACCACTCATAAAA |
| PBK | TCGGGACCAATTTTGAAGAG | GCCCAAGGAGAATGAGACAA |
| CEP55 | CAGTATCCAGCCACTGAGCA | GGGAGGTATCACTGCCAAGA |
| RRM2 | ACAGAAGCCCGCTGTTTCTA | CCCAGTCTGCCTTCTTCTTG |
| ASPM | GAGGCTGGAAAGCATGAAAG | AGCATTCTTCACAGCCAGGT |
| NCAPG | TTCAAGGCTGGTTACGGTTC | AGGGCACACCAATACAAAGC |
| CDKN3 | CATAGCCAGCTGCTGTGAAA | CCCGGATCCTCTTAGGTCTC |

**Table S2 Top 15 up and down-regulated differentially expressed genes in GSE9452 dataset**

| **Name** | **Description** | **log2FoldChange** | **pval** | **up/down** |
| --- | --- | --- | --- | --- |
| REG1A | regenerating family member 1 alpha | 8.19 | 5.34E-12 | **up** |
| REG1B | regenerating family member 1 beta | 7.37 | 2.71E-08 | **up** |
| REG3A | regenerating family member 3 alpha | 7.17 | 1.97E-10 | **up** |
| DEFA5 | defensin alpha 5 | 6.18 | 1.11E-08 | **up** |
| CHI3L1 | chitinase 3 like 1 | 5.71 | 4.03E-08 | **up** |
| S100A8 | S100 calcium binding protein A8 | 5.39 | 2.37E-08 | **up** |
| LCN2 | lipocalin 2 | 5.28 | 1.88E-11 | **up** |
| SLC6A14 | solute carrier family 6 member 14 | 5.19 | 3.09E-08 | **up** |
| DUOX2 | dual oxidase 2 | 5.12 | 4.58E-10 | **up** |
| MMP3 | matrix metallopeptidase 3 | 4.97 | 6.24E-07 | **up** |
| CXCL1 | C-X-C motif chemokine ligand 1 | 4.87 | 3.17E-12 | **up** |
| PI3 | peptidase inhibitor 3 | 4.49 | 1.72E-09 | **up** |
| KYNU | kynureninase | 4.41 | 1.43E-08 | **up** |
| CXCL8 | C-X-C motif chemokine ligand 8 | 4.38 | 8.10E-08 | **up** |
| CXCL3 | C-X-C motif chemokine ligand 3 | 4.28 | 7.71E-11 | **up** |
| CA1 | carbonic anhydrase 1 | -2.93 | 1.75E-03 | **down** |
| MT1M | metallothionein 1M | -2.99 | 2.87E-03 | **down** |
| TRPM6 | transient receptor potential cation channel subfamily M member 6 | -3.04 | 1.26E-04 | **down** |
| GUCA2B | guanylate cyclase activator 2B | -3.06 | 6.12E-06 | **down** |
| GUCA2A | guanylate cyclase activator 2A | -3.16 | 1.92E-06 | **down** |
| SLC26A2 | solute carrier family 26 member 2 | -3.23 | 3.08E-05 | **down** |
| ABCB1 | ATP binding cassette subfamily B member 1 | -3.46 | 1.18E-08 | **down** |
| SIAE | sialic acid acetylesterase | -3.6 | 9.23E-13 | **down** |
| SLC16A9 | solute carrier family 16 member 9 | -3.72 | 9.32E-06 | **down** |
| UGT2A3 | UDP glucuronosyltransferase family 2 member A3 | -3.75 | 1.36E-06 | **down** |
| GHR | growth hormone receptor | -3.9 | 2.32E-11 | **down** |
| PNLIPRP2 | pancreatic lipase related protein 2 (gene/pseudogene) | -3.99 | 8.95E-08 | **down** |
| PCK1 | phosphoenolpyruvate carboxykinase 1 | -4.36 | 1.17E-06 | **down** |
| AQP8 | aquaporin 8 | -4.46 | 3.67E-04 | **down** |
| CLDN8 | claudin 8 | -5.47 | 3.07E-09 | **down** |

**Table S3 Top 15 up and down-regulated differentially expressed genes in GSE53306 dataset**

| **Name** | **Description** | **log2FoldChange** | **pval** | **up/down** |
| --- | --- | --- | --- | --- |
| REG1B | regenerating islet-derived 1 beta | 4.73 | 3.65E-10 | **up** |
| SAA2 | serum amyloid A2 | 3.97 | 1.36E-09 | **up** |
| DUOXA2 | dual oxidase maturation factor 2 | 3.69 | 1.67E-14 | **up** |
| REG1A | regenerating islet-derived 1 alpha | 3.63 | 1.10E-06 | **up** |
| MMP3 | matrix metallopeptidase 3 | 3.54 | 1.20E-12 | **up** |
| REG3A | regenerating islet-derived 3 alpha | 3.52 | 4.28E-05 | **up** |
| SPINK4 | serine peptidase inhibitor, Kazal type 4 | 3.45 | 3.49E-08 | **up** |
| SLC6A14 | solute carrier family 6 | 3.43 | 2.49E-13 | **up** |
| PI3 | peptidase inhibitor 3, skin-derived | 3.32 | 2.15E-12 | **up** |
| LCN2 | lipocalin 2 | 3.28 | 1.28E-07 | **up** |
| KRT6B | keratin 6B | 3.06 | 4.65E-07 | **up** |
| NOS2A | nitric oxide synthase 2A | 3.01 | 4.92E-07 | **up** |
| DMBT1 | deleted in malignant brain tumors 1 | 2.98 | 1.29E-11 | **up** |
| DUOX2 | dual oxidase 2 | 2.96 | 2.00E-10 | **up** |
| CLDN2 | claudin 2 | 2.95 | 2.89E-08 | **up** |
| PGAM4 | phosphoglycerate mutase family member 4 | -2.59 | 6.30E-11 | **down** |
| CREB3L3 | cAMP responsive element binding protein 3-like 3 | -2.66 | 4.74E-07 | **down** |
| CLDN8 | claudin 8 | -2.70 | 2.26E-09 | **down** |
| PXMP4 | peroxisomal membrane protein 4, 24kDa | -2.70 | 8.53E-08 | **down** |
| PRAP1 | proline-rich acidic protein 1 | -2.71 | 1.06E-06 | **down** |
| SLC37A2 | solute carrier family 37 | -2.84 | 2.23E-05 | **down** |
| PLA2G12B | phospholipase A2, group XIIB | -2.86 | 4.70E-08 | **down** |
| ALDH1A3 | aldehyde dehydrogenase 1 family, member A3 | -2.90 | 3.38E-06 | **down** |
| AQP7 | aquaporin 7 | -3.00 | 4.02E-10 | **down** |
| TMIGD1 | transmembrane and immunoglobulin domain containing 1 | -3.03 | 2.61E-07 | **down** |
| GUCA2B | guanylate cyclase activator 2B | -3.10 | 4.84E-09 | **down** |
| ABCG2 | ATP-binding cassette, sub-family G | -3.13 | 6.47E-13 | **down** |
| SLC13A2 | solute carrier family 13 | -3.25 | 1.59E-10 | **down** |
| CNTFR | ciliary neurotrophic factor receptor | -3.45 | 6.00E-10 | **down** |
| AQP8 | aquaporin 8 | -3.54 | 6.65E-07 | **down** |
