# Supplementary material for: Knockdown of TOP2A suppresses IL‐17 signaling pathway and alleviates the progression of ulcerative colitis
Source: Immun Inflamm Dis. 2024 Apr 25;12(4):e1207. doi: 10.1002/iid3.1207 (PMC11044219; doi:10.1002/iid3.1207)
Supplement: Supplementary file 3 — Supporting information. [file IID3-12-e1207-s001.docx]

**Fig.S1** Box line plot. The correction results of GSE9452 and GSE53306 dataset samples.

**Fig.S2.** Venn diagram of common differentially expressed genes (DEGs). The sum of the numbers in each circle represents the total number of DEGs in the dataset, and the overlapping part of the circle represents the common DEGs between different datasets.

**Fig.S3** Enrichment analysis of differentially expressed genes. A. Bubble diagram of GO enrichment analysis. B. Bubble diagram of KEGG enrichment analysis. The color depth of the node represents the corrected P value, and the size of the node refers to the number of genes involved.

**Fig.S4** ROC curve analysis of NDC80, PBK, CEP55, RRM2, ASPM, NCAPG, TOP2A, CDKN3. The abscissa is false positive rate (FPR), the ordinate is true positive rate (TPR), and the expression value of the expressed gene in the sample data.
